# ‘Know that You are not Alone.’ Influences of Social Support on Youth Newly Diagnosed with HIV in Kibera, Kenya: A Qualitative Study Informing Intervention Development

**DOI:** 10.3390/ijerph16050775

**Published:** 2019-03-04

**Authors:** Nicole M. Lockwood, Kathryn Lypen, Firas Shalabi, Manasi Kumar, Elizabeth Ngugi, Gary W. Harper

**Affiliations:** 1 Department of Health Behavior and Health Education, University of Michigan School of Public Health, Ann Arbor, MI 48109, USA; lockwoni@umich.edu (N.M.L.); kdlypen@gmail.com (K.L.); fshalab@gmail.com (F.S.); 2 Department of Psychiatry, University of Nairobi, Nairobi 00202, Kenya; m.kumar@ucl.ac.uk; 3 Department of Clinical Health and Educational Psychology, University College London, London WC1E 7HB, UK; 4 Centre for HIV Prevention and Research, University of Nairobi, Nairobi 00202, Kenya

**Keywords:** HIV/AIDS, psychological and social barriers, phenomenological research, sub-Saharan Africa, perceptions of Kenyan youth

## Abstract

The role of social support in assisting youth in developed countries cope with their HIV diagnosis has been examined through a vast body of research; yet, there remains a gap in research around the effects of social support among youth living in sub-Saharan African countries including Kenya. This study aimed to examine the role of social support among Kenyan youth living with HIV, specifically with regard to the variations in influences of this social support. We conducted semi-structured focus group discussions with youth (ages 18 to 27) living in the informal urban settlement of Kibera in Nairobi, Kenya (*n* = 53). Data analysis followed a phenomenological inquiry framework, and seven major categories of perceived social support influences were identified: (1) linkage to services, (2) antiretroviral (ARV) adherence, (3) self-acceptance of HIV status, (4) healthy and positive living, (5) understanding of what it means to be living with HIV, (6) HIV status disclosure, and (7) family and occupational strengthening. The findings from this study suggest that Kenyan youth living with HIV can benefit from social support in a multitude of ways and can occur across several socio-ecological levels. Future research should further examine these influences, specifically regarding intervention development across socio-ecological levels.

## 1. Introduction

The global burden of HIV remains high: there were 36.9 million people living with human immunodeficiency virus (HIV) in 2017 [1]. Kenya has been more affected by HIV than have many other parts of the world, with prevalence rates within the top fifteen highest country-level rates; UNAIDS estimates that, in 2017, 1.5 million Kenyans were living with HIV [2,3]. That same year, HIV was the leading cause of disability adjusted life years (DALYs) and of death in Kenya [4]. At 6.1%, the adult HIV prevalence rate in Nairobi is higher than Kenya’s country-level rate of 4.9% and considerably higher than the global rate of 0.8% [1,5].

Prevalence rates in Kenyan informal urban settlement areas, such as Kibera—Nairobi’s most populated urban informal settlement—are estimated to be even higher, at 12% [6]. Several factors, including poverty, unemployment, and substance abuse, make living in Kibera a high-risk environment for transmission of HIV [7]. Women may be at even higher risk; as a study by Amuyunzu-Nyamongo et al. (2017) found, women living with HIV in Nairobi informal settlements had commonly taken to survival strategies such as commercial sex work, which placed them at significant risk for HIV reinfection and infection with sexually transmitted infections (STIs) [8].

HIV continues to disproportionately impact Kenyan youth, with incidence rates highest among those nearing adulthood [9]. A disproportionate share of incident adult HIV infections in Nairobi County occur among youth aged 15–24: in 2016, this population accounted for 46% of all new HIV infections in Nairobi County yet just 18% of the Nairobi County population [10]. In 2017, HIV was the leading cause of DALYs and of death among Kenyans aged 10–24 years old [4]. Combined with the general HIV rates in informal urban settlements, youth living in such areas represent a group highly vulnerable to the negative effects of HIV.

In the initial period after receiving an HIV diagnosis, youth can undergo particularly high amounts of emotional stress. Those who test positive for HIV are not only burdened with adjusting to living with a highly stigmatized health condition that requires lifelong medical management but are also often struggling with typical adolescent developmental challenges [11,12]. This stress can primarily be attributed to adjusting to their diagnosis and disclosing their HIV status to others, which is often compounded by lack of accurate information and fears related to HIV medications and development of symptoms [11].

As a result, youth living with HIV often experience reduced quality of life, psychological distress, social impairment, increased risk-taking behaviors, poorer antiretroviral (ARV) medication adherence, and increased exposure to additional psychosocial vulnerabilities [11,12,13]. Furthermore, the stigma associated with HIV may serve as a barrier to youth acquiring or maintaining social support [14]. As such, there is need for new, targeted interventions specifically for youth in the initial period after receiving an HIV positive diagnosis.

Social support, based on pivotal work by House (1981), comprises providing emotional, instrumental, informational, or appraisal aid and assistance within social relationships [15]. Numerous studies from developed countries have found that social support positively impacts health and wellbeing, including with improving overall health, improving stress response, and improving coping related to HIV management [16,17,18]. Though diverse in their specific objectives and sample characteristics, there have been several qualitative studies among people living with HIV in sub-Saharan Africa that describe positive impacts from social support [19,20,21,22,23,24]. However, there remains a gap in such research among youth living in a large informal urban settlement like Kibera, as well as research specifically targeting the initial stage after being diagnosed with HIV. Moreover, none of these studies examined as deeply the diverse influences of social support received across informal and formal sources without some sort of scope or other limitation.

Youth remain largely neglected when it comes to targeted service provision in Kenya [25,26,27]. To develop interventions that successfully address the unique barriers Kenyan youth may face in dealing with a recent HIV diagnosis, it is imperative to understand their lived experiences. Therefore, the purpose of this study is to better understand the diverse perceived influences of social support among youth newly diagnosed with HIV (i.e., within the past two years) living in urban informal settlements in Kenya, as it impacts their HIV management and other aspects of their lives.

## 2. Materials and Methods

### 2.1. Data Collection

To understand the role of social support within the experiences of Kenyan youth newly diagnosed with HIV, six focus group discussions were conducted with youth residents of Kibera, the informal urban settlement in Nairobi. Qualitative data was collected through focus group discussion (FGD), as the aim of the study was to gain a phenomenological understanding of the lived experiences of youth newly diagnosed with HIV, and the group dynamic could provide potentially richer data than could individual interviews with regard to the phenomena of interest [28]. In a qualitative study by Hosek et al. (2008) in the United States with youth newly diagnosed with HIV, data were collected through both FGD and in-depth interview (IDI), and the researchers found that the group dynamics in the FGD helped participants to build off of each other’s comments and aided recall by triggering thoughts and memories that assisted participants in sharing greater detail [11].

Given that the data presented in this paper are part of a larger study investigating risk and resiliency, Wallander and Varni’s (1992) Disability-Stress-Coping Model served as an initial framework for the focus group discussions to determine both the key sources of stress (i.e., psychological stress, functional independence, disease parameters) and coping mechanisms (i.e., intrapersonal competence, socio-ecological support, coping strategies) following each participant’s HIV diagnosis [29]. Semi-structured interview guides, based on a phenomenological framework, were developed with open-ended questions to investigate these key areas.

‘Newly diagnosed’ was defined as having received an HIV diagnosis within the past two years and focused on the first six months following diagnosis. The time period was selected in consultation with HIV service providers and youth living with HIV in Kenya regarding how to capture initial challenges and opportunities the youth faced with managing their HIV. A chain-referral sampling technique was used to recruit a purposive sample of youth through existing peer networks, as peer-referrals allowed access to the predominantly-hidden group of youth living with HIV. Participants represented eight ethnic groups, as efforts were made to recruit youth from diverse ethnic groups. Focus group discussions held in Kibera lasted between two to three hours, with a total of 53 participants (aged 18–27) from ten villages in Kibera. Though youth is often defined as those aged 15–24, Kenyan policy often refers to youth as 15–30 [30]. The age range of study participants reflects the culturally appropriate definition of youth.

Recruited individuals needed to meet the following inclusion criteria to participate: (1) be aged 18–27 years old, (2) have been infected with HIV through a behavioural mode of transmission, (3) have an initial HIV diagnosis date that falls within the past 24 months, (4) be willing to participate in focus group discussions, and (5) provide informed consent for study participation. Exclusionary criteria for participation included those individuals who (1) acquired HIV through perinatal infection, (2) demonstrated or reported any presence of serious psychiatric symptoms, (3) were visibly distraught, or (4) were intoxicated or under the influence of alcohol or other substances at the time of study enrolment. None of the originally recruited participants were excluded based on these criteria.

Participants completed a demographic information survey prior to participation in the focus group. Bilingual local research team members facilitated focus groups in both English and Kiswahili, based on participant preference. Focus groups were gender-specific in order to promote more open dialog that was less restricted by gender norms and related social power dynamics, particularly given the stigma around the discussion topic. Each gender-specific focus group (three groups of women and three groups of men) consisted of 8–10 participants. A member of the local research team transcribed/translated into English the digitally-recorded focus group discussions.

The local research team had prolonged engagement with the participant population through various programs in the field in order to assess possible sources of data distortion and to identify saliencies in the data. Through a continuous informal testing of information, participants’ reactions to data as they were being collected were solicited in order to assess the accuracy of the investigators’ reconstructions of what was being said. Our research team included individuals with various disciplinary backgrounds (e.g., Nursing, Public Health, Clinical Psychology, Community Psychology, and Social Work), as well as different countries of origin (Kenya and United States). These various disciplinary and geographic backgrounds allowed for investigator triangulation throughout the study.

### 2.2. Data Analysis

We conducted data analysis using a phenomenological inquiry framework. The composite descriptions discussed in this article describe both what and how phenomena were experienced by respondents [28,31]. We identified patterns related to the primary concepts being explored, specifically regarding the perceived influences of receiving social support by Kenyan youth newly diagnosed with HIV, and developed inductive codes through examining the data. Social support was operationalized as aid and assistance provided within social relationships, in accordance with the fundamental work by House [15]. We added subsequent codes during the iterative analysis process.

We added supplemental content codes to the code list based on the lived experiences described by participants after reading and reviewing all transcripts and developed a codebook that included operational definitions of all codes. Pattern codes were then assigned to link related concepts together, after which we established consistent patterns in meaning, concepts, and themes across all focus group transcripts [28,32]. All codes were developed and independently assigned by the first three authors, who resolved any inconsistencies in data interpretation and pattern identification through discussions leading to unanimous agreement. Findings were shared during data analysis with professional peers who were not actively engaged in the study in order to receive external feedback on the analysis process, as well as to test and clarify interpretations of the data [33,34]. This article focuses on the identified theme of social support influences that emerged during the focus group discussions.

### 2.3. Ethical Considerations

Institutional review board approval was obtained from all participating institutions for the research protocol (GH071505PSY). Written informed consent was obtained from each participant prior to their involvement in a focus group. Youth received reimbursement for any transportation costs and a meal to compensate them for their time. Throughout the data collection and analysis process, the research team protected participant confidentiality. To protect patient identities, transcripts were de-identified for analysis and pseudonyms were assigned to participants.

## 3. Results

A total of 53 youth (49.1% women; 50.9% men) participated in this study’s focus group discussions. Table 1 summarizes demographic characteristics of study participants. Participants were aged 18 to 27 years old at the time of the study, and the majority were unmarried.

Study participants reported having received social support within their first six months after diagnosis, which they perceived as having diverse influences. This social support fell into four major categories, aligning with the four major types of supportive actions categorized by House: instrumental support, defined as receiving something tangible that helps remove barriers youth may encounter; informational support, defined as receiving advice, factual knowledge, or suggestions that enables the adolescent to cope with adversity; emotional support, defined as receiving comfort, empathetic listening, or consolation that helps the adolescent cope with emotional distress; and appraisal support, defined as receiving constructive feedback, assurance, or validation that enables the adolescent to better adjust to his or her situation [15].

While several participants described times of feeling isolated, many reported positive experiences related to having received social support during the initial six months after their HIV diagnosis. These perceived social support influences fell into seven domains: (1) linkage to services, (2) ARV adherence, (3) self-acceptance of HIV status, (4) healthy and positive living, (5) understanding of what it means to be living with HIV, (6) HIV status disclosure, and (7) family and occupational strengthening. There were also some instances in which respondents described social support influences as positive in too general of a sense to ascribe to one of the previously outlined categories. These seven main perceived influences of social support indicate that social support could diversely benefit Kenyan youth newly diagnosed with HIV. Below, we describe the details of each thematic influence of social support, and sub-themes that form the larger influences of support. The speaker’s gender and age follow any text quoted from participants.

### 3.1. Linkage to Services

Youth participants recalled that the social support they received in their first six months post-diagnosis had the perceived effect of linking them to HIV services, including integrated psychosocial and clinical care. The internalization of the diagnosis and its long-term import in terms of medication and regular association with the health facility was of importance here. Two major sub-themes emerged under this theme: (a) connecting with a health center, counsellor, or support group and (b) connecting with ARV medication.

#### 3.1.1. Connecting with a Health Center

Participants described how support they received aided them in connecting with a health center, counsellor, or support group. The initial support paved the way for a more nuanced socio-emotional mental health follow up with a specialist. Anna recalled her disbelief that she could have a positive HIV diagnosis, which prevented her from accepting her status and taking steps to manage the disease. She eventually visited another doctor who went above and beyond that of his or her duties:

*‘I felt sick but, at the back of my mind, I knew I had been told that I am HIV positive. During this time, I decided to go to the doctor; lucky enough, I found a good doctor who asked why I had allowed the disease to attack me to this extend* [sic]. *I told the doctor that, still, I don’t believe [I] am sick. The doctor took me to another doctor who tested me but, still, the results were the same’*.

(25, woman)

#### 3.1.2. Connecting with ARV Medication

A 25-year-old man, Thabiti, described how the social support he received connected him with ARV medication. Thabiti affirmed the challenges in obtaining ARVs due to inconsistent supply volumes and fatigue from waiting in the long lines at the ARV clinic, as others in the focus group had described. Thabiti’s parents enacted support by picking up the drugs for him. He was able to obtain his ARV medication due to his parents’ support.

### 3.2. ARV Adherence

Youth reported that social support they received helped them adhere to their ARV regimens by addressing challenges associated with ARV initiation or continued adherence. This influence type does not involve physically connecting the youth with ARVs. Two sub-themes emerged: (a) addressing challenges to adherence and (b) building adherence skills.

#### 3.2.1. Addressing Challenges to Adherence

A few participants perceived that external support alleviated difficulties they faced in remembering when to take their medications. These difficulties were assuaged through receiving reminders, receiving information regarding the importance of never missing a dose, or becoming motivated to adhere through encouragement. When participants reported receiving motivational and supportive care, they perceived that their illness burden reduced. Hadiya had difficulty remembering when to take her ARV medications:

*‘I had problems the first month, but I had a doctor who cared for me. He reminded me when to take drugs for the first three month. We had agreed when I should take my medicine, so when it was time, he call* [sic] *or sent me a please call me, then I know it’s my time to take my ARVs. I continued like that until I can remember on my own’*.

(25, woman)

#### 3.2.2. Building Adherence Skills

Another sub-theme discussed involved social support the youth received resulting in them learning a new skill that made adhering to their prescribed drug regimens more feasible in the face of HIV-related stigma. Through a support group, Faith learned to store her pills safely without fear of accidental disclosure:

*‘Disposing of the empty bottles of drugs was a challenge initially. That time I would go with a polythene bag and put all the drugs there and leave the container in the hospital. I later came to learn it was not good. The moment I joined the support group, I gained the knowledge of removing the labels on the bottles and disposing became a little bit easily* [sic]’.

(25, woman)

### 3.3. Self-Acceptance of HIV Status

Many participants discussed gaining self-acceptance of one’s HIV status due to having received social support. Prior to this self-acceptance, participants described experiencing self-stigma and developing internalizing tendencies that led to self-doubt and poor self-efficacy to manage their HIV and live healthily. This social support influence often involved someone directly telling them that they should accept their status or providing examples of other young people living with HIV. Fadiya explained how an interaction with a close friend helped change her perception of what it meant to be a person living with HIV:

*‘I really had stress. After that, she called me and told me that she was also a victim, but when you look at her, you can’t believe it, she is healthy and big. At that moment, that is when I began to regain myself’*.

(24, woman)

Similarly, Inaya shared that her husband’s parents aided her in accepting her HIV status:

*‘My husband’s parents who consoled me that I was not the only one suffering, I should accept my situation and, indeed, I accepted’*.

(21, woman)

### 3.4. Healthy and Positive Living

The participants described that social support they received also had the perceived effect of helping them to live positively, outside of making healthy choices directly related to managing their HIV diagnosis. We identified two sub-themes under this influence: (a) improving physical health and (b) improving emotional/psychological wellbeing.

#### 3.4.1. Improving Physical Health

Several youth participants perceived social support they received or desired as helping them with their opportunistic infections or with another physical ailment. For example, Thabiti, age 25, had an accident and desired that his parents provide him with social support by donating blood.

#### 3.4.2. Improving Emotional/Psychological Wellbeing

Another sub-theme associated with positive living was an improvement in participants’ emotional or psychological wellbeing as they received support. Participants spoke about various ways in which their emotional/psychological wellbeing was bettered. This included building new skills, releasing stress, practicing religious faith, and connecting with others. Azizi described the support he received from his friends:

*‘Emotionally, I was so down; like, I was finished, my life was coming to an end. I started drinking heavily, spending a lot of money anyhow, I had not* [sic] *future […] I came to realize that am not the only one in this situation, so I got some friends whom we attended therapy with together* [sic], *and that helped me heal my emotional problems’*.

(23, man)

### 3.5. Understanding of What It Means to be Living with HIV

Many youth participants revealed that the social support they received altered their understanding of what it means to be living with HIV in two distinct ways: a) acknowledging they are not alone in facing their HIV diagnosis and b) believing it is possible for people living with HIV to live a long and healthy life.

#### 3.5.1. Acknowledging They are not Alone in Facing Their HIV Diagnosis

Some youth recalled feeling a strong sense of isolation after receiving a positive HIV diagnosis. Specifically, they believed that they were alone in their struggle. Social support had an impact on altering this belief. In these cases, the social support was commonly from other youth living with HIV. For Kwasi, it was his peers who facilitated his change in understanding:

*‘You find one youth who is really sick but the morale they have just gives you psychic* [sic] *also to continue with life. You say, for sure, if this guy has made it in life, why not me. So that counselling sessions we have together encourages you at least to know that you are not alone’*.

(18, man)

#### 3.5.2. Believing It is Possible for People Living with HIV to Live a Long and Healthy Life

When first learning of one’s HIV diagnosis, some youth believed HIV was a death sentence. In these instances, youth often indicated feeling relieved, yet surprised, when they encountered another person living with HIV who appeared strong and healthy. Such encounters helped youth to better understand that one can be in good health while living with HIV. For Kafil, counselling helped him realize this:

*‘With time, I accepted myself. I went through counselling where I was informed that this disease was like any other disease in human life as long as you adhere to the rules given to make the disease sustainable’*.

(27, man)

### 3.6. HIV Status Disclosure

Youth described how the social support they received allowed them to disclose their HIV status to someone, often a family member or friend. Two sub-themes were identified under this theme: (a) building confidence and/or skills to disclose and (b) receiving direct assistance with disclosure.

#### 3.6.1. Building Confidence and/or Skills to Disclose

For some participants, the social support they received provided them with the confidence and/or skills needed to disclose his or her status to another. This could involve providing encouragement, reassurance, a safe space, or skills to use in order to disclose. Kibibi described:

*‘Initially, I feared to tell him for a period of six months […] Through the teaching I received from the clinic, one day I decided I will tell [my husband] even if it means him sending me away’*.

(21, woman)

#### 3.6.2. Receiving Direct Assistance with Disclosure 

Several of the youth participants shared experiences of another individual providing them with direct assistance around disclosure. A friend helped Inaya when the friend disclosed Inaya’s status to her husband’s parents, who in return, provided her with additional social support that helped her to accept her HIV status and adhere to her ARV medication:

*‘I don’t have parents except my husband’s parents […] I didn’t tell the parents of my husband. For a whole week, I didn’t eat; I was just quiet. A friend came who asked me what the problem was and I explained to her, she late* [sic] *went and told my husband parents’*.

(21, woman)

### 3.7. Family and Occupational Strengthening

Participants also described that the social support they received helped them cope with aspects of their lives not directly related to HIV. Two main sub-themes were identified under this theme: (a) strengthening family/relationships and (b) improving job or economic stability.

#### 3.7.1. Strengthening Family/Relationships 

A few participants recounted how the social support they received strengthened their family and/or other relationships. In these instances, participants felt their romantic relationship or family stability—such as with a spouse or partner—was insecure and may soon end. However, receiving social support strengthened and stabilized the youth’s unsteady relationship.

#### 3.7.2. Improving Job or Economic Stability

Participants also reported receiving support that allowed them to continue working at their place of employment or that validated their participation in income-generating activities. Kafil feared that his boss was going to fire him due to his HIV positive status. He thought this because his position required him to travel and his HIV status could prevent him from obtaining a passport:

*‘I went back to the office, informed my boss who took time to think over it, but later, he allowed me to continue with my job; this gave me morale and courage of moving on with life’*.

(27, man)

## 4. Discussion

This study explored the effects of social support perceived by Kenyan youth newly diagnosed with HIV. The phenomenological research framework allowed for an in-depth understanding of the diverse influences of social support experienced by these youth. This social support, provided in the forms of instrumental, informational, emotional, and appraisal support, impacted their HIV management and other realms of their lives. Study participants discussed experiencing seven main effects of social support. The support came from both formal and informal sources. Formal support sources included health facility staff, such as clinicians, counsellors, and support groups, as well as non-governmental organizations. Informal sources included family members, friends, spouses/romantic partners, participants’ beliefs in religious practices (e.g., praying, speaking with a pastor), neighbors, and bosses. For many participants, social support played an integral role in strengthening their sense of resolve to persist in life despite the diagnoses of HIV.

Our data, in-line with previous scholarship, indicate that social support can positively influence Kenyan youth newly diagnosed with HIV [18,20,21,35,36]. These social support influences can be far-reaching and can occur across several socio-ecological levels, consistent with the findings of Hosek et al. (2008), which suggest youth recently diagnosed with HIV experience stressors within multiple social-ecological systems [11]. Participants in our study described what appeared to be factors enabling them at multiple tiers, including at the individual, interpersonal, and organizational levels. Physical health was improved at the individual and organizational levels, as described by the themes of linkage to services, ARV adherence, and healthy and positive living. Our participants shared that their mental health and wellbeing were also improved after receiving social support at these levels. This was achieved through self-acceptance of one’s HIV status, understanding of what it means to be living with HIV, and family and occupational strengthening. At the interpersonal level, social support aided participants in disclosing their HIV status to others or through strengthening relationships. Though the study focused on the direct influences of perceived social support, there are also many indirect influences. For example, for youth struggling with disclosing their HIV status to others, self-acceptance may facilitate a greater sense of self-efficacy for a successful disclosure event. Furthermore, participants did not describe any instances in which receiving social support had a negative impact. Our inquiry supports the notion that social support is a strong effect modifier and might significantly improve positive living and thinking among youth newly diagnosed with HIV.

Similar studies of youth living in Tanzania and Botswana and adults living in South Africa, Swaziland, Uganda, and Kenya broadly describe influences of support that are similar to the themes elaborated by our participants during the focus groups—though, not necessarily using the same terminology nor as much detail as in this paper [19,20,22,23,24]. These studies largely focused on a few influences directly related to HIV, such as reporting decreases in felt stigma, better adherence to ARV regimens, and/or improvements in coping. The influences of receiving social support as described by participants in our study reinforce the positive social support effects described by South African youth in a 2010 study by Petersen et al. This study demonstrates that social support is important for coping and general well-being among adolescents living with HIV [21].

This study is unique given that the influences of social support were operationalized from an unrestricted set of sources that, when coupled with the phenomenological research framework, elicited a wide range of influences not often considered. Other studies of this nature did not limit participant inclusion to those newly diagnosed with HIV. Exclusively examining the initial period after diagnosis provides insight into the role of social support during this period of high emotional stress experienced while adjusting to one’s diagnosis [11]. This study expands the current body of research by offering a deeper and broader understanding of the impact that received social support can have among youth newly diagnosed with HIV in Kenya and, potentially, in other sub-Saharan African countries. Furthermore, this study looks beyond the social support influences that directly impact the youth’s HIV management, recognizing that their HIV status does not define their complete lived experience and sense of self.

The findings from this study emphasize the key role of incorporating social support into future secondary and tertiary prevention efforts designed to aid youth living with HIV in coping with their diagnosis. Not all study participants described having received social support during their first six months after diagnosis, while others were isolated for a shorter period after diagnosis. These avenues for intervention development involve taking a socio-ecological approach and including multiple stakeholders. This is particularly important given the barriers that individuals living with HIV can face in accessing these formal sources of support, such as barriers for men to participate in support groups as described in the 2012 study by Madiba and Canti-Sigaqa [37]. For instance, at the individual level, health providers may benefit from targeting social support sources in their interventions to improve ARV adherence and/or understanding of the disease. Kulzer et al. (2012) describe an innovative provider-initiated engagement strategy called the family model of care, a tool specifically designed to build upon the strengths of Kenyan families. In this model, providers use a family information table (FIT) to guide people living with HIV through the steps of identifying family members who may be at risk for HIV. At the same time, providers also address concerns related to status disclosure and facilitating family testing. Starting at the individual level, this model encourages each new patient to consider themselves as part of a group at risk for HIV and recognize that they do not have to face their diagnosis alone [38]. Comprehensive programs such as this could play a significant role in helping Kenyan youth better understand the implications of living with HIV. Such programs could also promote the family unit as a source of continued support from which additional influences of social support could arise.

At the dyadic level, for example, an individual may better adjust to their partner’s new HIV diagnosis if interventions in which the sero-discordant couple participates demonstrate how to provide social support to one another. In a recent study of 469 HIV sero-discordant couples in Nairobi, incidence of relationship dissolution was high, with 24% of couples reporting separation during a two-year follow-up period. In the study, separation was most common among partners of low socioeconomic status and for women living with HIV [39]. For youth living in a high-risk environment such as Kibera, programs designed to maintain stability among sero-discordant partners could play an important role in both HIV prevention efforts and improving the health of the partner living with HIV. Moreover, social support and partner-focused interventions may lead to better engagement in treatment and care, utilization of counselling and testing services, and improved ARV adherence [23,39,40].

At the organizational level, a social support intervention could initiate a paradigm shift in how an organization supports their members living with HIV. Such an intervention could potentially result in positive dissemination effects for other organization members, due to the connection of social support to general health and well-being [16,17,41]. For instance, religious organizations to which people living with HIV already belong could provide support to their members living with HIV, as described by Root (2009) and Watt et al. (2009). However, people living with HIV often reported feeling stigmatized during church participation [22,42]. Root (2009) found that, by defining personhood for people living with HIV, churches could assist members living with HIV with disclosure and help-seeking behaviors [22]. Utilizing faith-based organizations to address HIV stigma mitigation for young people living with HIV needs further exploration. Social support interventions among pre-existing organizational networks could counter stigma, encourage disclosure, promote HIV treatment, and impact wellbeing outside of HIV management.

Given the complex relationships among factors influencing health status, lasting and widespread impacts could result from strategically harnessing social support at multiple socio-ecological levels in interventions for youth living with HIV in Kenya. One mechanism by which this may be accomplished is through the use of social media platforms, including social networking sites and mobile technology. As the use of social media becomes more widespread, particularly among adolescent populations worldwide, interventions designed to leverage such forums could prove to play a pivotal role in the way information about—and experiences of those affected by—HIV is shared among individuals, peer networks, and communities at large. A meta-analysis examining the role of social media in HIV communication cited the vast benefits of using social media for this purpose, primarily as perceived by the target populations. These benefits included access to information, strengthened ability to communicate, allowing for anonymity, enhanced social and emotional support, forming a virtual community, and geographical reach. Adolescents in these studies reported experiencing a sense of community from connecting with others through social media [43]. For Kibera youth newly diagnosed with HIV, use of social media platforms such as SMS messaging or Facebook could allow them to connect virtually with others who may not be in their existing peer networks and provide opportunities to discuss topics that may be uncomfortable to broach in-person. Furthermore, the option to retain one’s anonymity through the use of social media could help to alleviate stigma and fear around HIV and allow youth who may not otherwise seek social support a chance to share personal stories they may not feel able to in their offline environment.

Social support may be particularly important early after diagnosis, allowing youth to receive such benefits while undergoing the often challenging and complicated process of adjusting to their new diagnosis. Based on the findings in this study, interventions aiming to achieve any of the seven influences discussed in this paper could incorporate social support into their models. It is important to note that youth living in Kibera may already be receiving some social support. Interventions should further foster this concept, to grow and diversify the realized social support effects. The desired influence(s) may differ depending on the needs of the participants and the support they are already receiving. Strengthening social support that youth receive may holistically impact the youth, as demonstrated by the described influences of healthy and positive living, as well as family and occupational strengthening. Though current interventions seek to promote social support at a specific socio-ecological level to directly impact HIV management, our findings indicate that influences of social support can be more widespread and the concept itself may be multi-layered. Recognizing the full potential of social support could address gaps in current interventions and lead to lasting impacts.

### Strengths, Limitations, and Future Research

This is one of first studies to investigate the influences of social support among Kenyan youth newly diagnosed with HIV. The sample for this study included representation of young men and women living in a high-risk, low-resource area in Kenya. Furthermore, the phenomenological approach allowed for a deep exploration of social support influences, due to the open-ended discussions with the youth that were not biased by closed-response options.

There are some limitations with this study. Given that the transcripts only included the verbal discussions that occurred during the focus groups and the analysts for this article were not present during the discussions, experiential and situational cues could not be used to further inform and guide the analysis. Additionally, participants may have had similar experiences due to the chosen recruitment technique of chain referral sampling and due to the fact that all participants were currently receiving HIV-related services and living in Kibera. These youth participants may receive more frequent or impactful social support than those not receiving such health care. Finally, as the data collection occurred at a single time point without follow-up, changes in perceived social support over time may have been missed.

Future studies should include additional populations, such as those from large urban cities, rural areas, and other sub-Saharan countries, to investigate if the findings in this study also apply, particularly as related research in such areas is lacking. Given the gender disparity within the HIV epidemic and the social and cultural differences experienced by Kenyan women, future research should explore the role of gender in how social support impacts individuals. As participants in the study described social support influences that fell outside of the direct realm of HIV management, future research should view HIV intervention development with a holistic lens through which it is recognized that an HIV diagnosis does not define a person’s complete identity and the interconnectedness of factors impacting health status.

Mixed-method studies could provide a deeper exploration and more standardized and targeted information from study participants. Such studies could inform successful intervention development by illuminating key features of the support influences identified in this study. Additionally, a longitudinal study could explore how perceived support effects change over time. The order in which participants experienced different influences of support appeared to have varied by participant; however, this may have been due to reporting errors stemming from data collection occurring at a single time point.

## 5. Conclusions

This qualitative study provides detailed insight into the diverse influences that Kenyan youth newly diagnosed with HIV can experience by receiving social support. Receiving social support may help youth cope on multiple levels, from a socio-ecological perspective. As such, it could complement a wide range of interventions. More research is needed to better understand the relationship between social support and the different influences, as well as how best to elicit desired effects through interventions. With the growth of social media popularity and potential of digital networks to expand access to social support, future research should examine the role of this media to provide social support influences within these marginalized populations.

## Figures and Tables

**Table 1:** Demographic information of the study participants.

Demographics	Participants
**Age, µ (±)**	22.8 (±2.4)
**Ethnicity, *n* (%)**	
Luo	24 (45.3%)
Luyha	9 (17.0%)
Kamba	8 (15.1%)
Nubian	4 (7.5%)
Kikuyu	3 (5.7%)
Kisii	3 (5.7%)
Mumeru	1 (1.9%)
Teso	1 (1.9%)
**Marital status, *n* (%)**	
Unmarried	33 (62.3%)
Married	20 (37.7%)
